# Medical Opinion and Movement

**Published:** 1906-09-08

**Authors:** 


### Medical Opinion and Movement.

The Health Department of the London County Council is the one department which has been the least in evidence of late. The health authorities of New York have taken a new departure which we commend to the attention of Sir Shirley Murphy and the London County Council Committee. In New York a series of exhibitions have been organised in the parks and recreation centres to illustrate the ravages of tuberculosis and how to treat this disease and prevent infection. Stereoscopicon views have been largely availed of to make the exhibitions effective and interesting. The exhibitions are under the direction of Dr. John S. Billings, jun., and Dr. Walters, and we hope next season will see similar shows in the parks of the Metropolis as well as in large provincial centres.

Medical inspection of school children in London, organised under Sir William Collins's direction was a new departure full of promise. In Philadelphia, where school inspection was first commenced some years ago, the Society for the Prevention of Cruelty to Children succeeded in bringing the Board of Health, Board of Education, and the Court for Juvenile Delinquents into co-operation with themselves to secure thorough inspection of each child. In practice the Philadelphia system amounts to a clinical service for the continuous investigation of the bodily and the physical and mental conditions of school children and the enforcement of immediate and adequate treatment and classification. Some ten thousand children are dealt with in Philadelphia alone.

The phenomenal heat has made most people who have been able to spend their days in the country fully acquainted with the annoyance of insect pests. Dr. J. Hepworth states that the essential oil of citronella applied to the skin in very small quantities will prevent midges and biting flies even from settling on tne skin. Every resident in tropical regions has tried this in the form of rubbing over the irritated skin the juice and the rind of a fresh lime. They have yet to learn that it will prevent either midges, mosquitoes, or biting flies from doing injury. There is no doubt, however, that it is soothing and relieves the itching and irritation. This purpose is best served by Dr. Bengue's balsam, the application of which immediately allays all irritation and renders life endurable in districts where mosquitoes and other biting insects abound. It is comforting to the possessors of the most delicate and refined skin. The balsam mentioned can be had from all the usual sources.

The papers have contained a report of an inquest on a seven-months' infant weighing only 4 lbs. Dr. Freyberger stated that the death was due to malnutrition. The child had been fed on goat's milk, which, Dr. Freyberger is reported to have said, was worse than skim milk, and did not contain sufficient fat or sugar. Captain Blackham, R.A.M.C., has pointed out that some authorities show that, after cows' milk, goats' milk is the most nutritious which can be used for the feeding of children. It is, he alleges, useful especially in cases of children who cannot digest cows' milk. Captain Blackham shows that goats' milk is rich in all the constituents of a perfect food, and not to be compared to skim milk as a form of nourishment. But it is not found to be generally suitable for the infant's digestive organs. The casein forms into hard lumps or nodules in the infant's stomach resembling in consistency and shape marbles made from Gruyère cheese. The whole question of milk as a nutritive agent depends on its digestibility. Perhaps Captain Blackham has not had in the Army Medical Corps sufficient opportunities to watch the effect of milk on the nutrition of babies. Doubtless Dr. Freyberger could produce the pathological evidence necessary to support what all general practitioners and clinicians are sufficiently aware of—the unsuitability of goats' milk for infants and young children.

The Medico-Political Committee of the British Medical Association have issued their report advocating the amendment of the law relating to the detention and care of inebriates and others suffering from the habitual use of drugs. Being convinced of the urgent need for legislation providing for the detention under suitable control of persons afflicted with those habits whereby they may be restrained from excessive indulgence in alcohol or drugs, whether they are willing or not to be so controlled, and whether they have been previously convicted or not, the Committee recommend the passing of an Act to legalise such detention. They are of opinion that the following safeguards should be enforced by law. The consent of a judicial authority should be necessary. Such authority should be given power to give costs against any applicant for the detention order whose application ought not in their opinion to have been made. Before the restraining order is issued, in spite of the patients' objection thereto, evidence should be forthcoming to prove that the habitual use of alcohol, opium, or any stimulant, sedative, or narcotic drug or substance is such as (*a*) to render the patient at times a danger to himself and others; (*b*) to render him at times incapable of managing himself or his affairs. Draft regulations are submitted giving the judicial authority power to commit for three years, with power to the Secretary of State to transfer the inebriate from the control of any guardian in whose charge he may have been placed by order of the Court, where the treatment is not satisfactorily enforced. Power is taken to deal with the estates of all persons placed under such restraint, and a procedure is laid down to cover all matters which the mandate is drafted to enforce. The measure in practice should prove a boon to the community and a blessing to the victims of these noxious habits.

